# Editorial: Community series in research advances of tuberculosis vaccine and its implication on COVID-19, volume III

**DOI:** 10.3389/fimmu.2026.1757051

**Published:** 2026-01-13

**Authors:** Wenping Gong, Ashok Aspatwar, Jianping Xie, Hao Li

**Affiliations:** 1 Senior Department of Tuberculosis, The Chinese PLA General Hospital, Beijing, China; 2 Faculty of Medicine and Health Technology, Tampere University, Tampere, Finland; 3 Institute of Modern Biopharmaceuticals, State Key Laboratory Breeding Base of Eco-Environment and Bio-Resource of the Three Gorges Area, Key Laboratory of Eco-environment of Three Gorges Reservoir, Ministry of Education, School of Life Sciences, Southwest University, Chongqing, China; 4 College of Veterinary Medicine, China Agricultural University, Beijing, China

**Keywords:** COVID-19, immunology & infectious diseases, protection, tuberculosis, vaccine

## 1 Background

The relentless global burden of Tuberculosis (TB), causing over 10.7 million new cases and 1.25 million deaths in 2024, demands urgent innovation (1). The limited and variable efficacy (0-80%) of the *Bacille Calmette-Guérin* (BCG) vaccine in adults, a challenge starkly highlighted during the COVID-19 pandemic, underscores the critical need for novel prophylactic and therapeutic strategies (2–5). This third volume of our Research Topic, “Community Series in Research Advances of Tuberculosis Vaccine and its Implication on COVID-19,” collates pioneering work that bridges immunology, nanotechnology, and clinical research. The eight articles herein not only provide deep insights into TB immunopathogenesis but also present tangible advances in vaccine design, diagnostic precision, and patient stratification, with broader implications for managing intracellular infections.

## 2 Key advances in this Research Topic

This Research Topic comprises eight articles (4 Research Articles, 2 Reviews, 2 Corrections) that collectively address critical bottlenecks in TB immunology, vaccine development and immunodiagnostics (**Table 1**).

**Table 1:** Summary of eight articles published in this Research Topic.

No.	Article type	Authors	Key findings summary
1	Research Article	Yang Gong et al.	• Developed Tri-GalNAc-modified PLGA-PEG nanoparticles (TP/GPS) that enhance dendritic cell (DC) uptake by 26%.
			• Potently activated DCs, inducing high IL-12p70 (p<0.001), and robust, long-lasting cellular immune memory.
			• Significantly reduced bacterial load in lungs in an H37Ra challenge model (p<0.05), with protection comparable to BCG.
2	Research Article	Xichao Ou et al.	• Identified the E-M recombinant antigen as a superior candidate for tuberculin skin testing (TST).
			• Induced a strong DTH response in guinea pigs (induration of 18.3 ± 1.8 mm at 10μg/ml) with no cross-reactivity to BCG.
			• Preclinical assessment confirmed excellent safety and specificity, supporting its potential for clinical trials.
3	Research Article	Xiaofang Liu et al.	• Identified a 3-gene cuproptosis-related signature (ASPHD2, GK, GCH1) for PTB diagnosis with high accuracy (AUCs: 0.981, 0.928, 0.937).
			• The signature was linked to a significantly altered immune microenvironment in PTB patients.
			• GCH1 and GK expression significantly decreased after one month of treatment (p<0.05), suggesting their utility as therapy monitoring biomarkers.
4	Research Article	Masoud Mortezazadeh et al.	• LTBI prevalence was not significantly higher in cancer patients (27.1%) vs. controls (20.7%, P = 0.176).
			• However, LTBI was a strong predictor of mortality in cancer patients (OR = 3.28, P<0.001).
			• Each 1mm increase in TST induration was associated with a 6% increased risk of death (HR = 1.06, P = 0.001).
5	Review	Aishwarya Shaji et al.	• Systematically reviewed the TB vaccine pipeline, noting the variable efficacy of BCG (0-80%).
			• Highlighted promising candidates like M72/AS01E, which showed 54% efficacy against MTB infection in adults.
			• Emphasized that host genetics (e.g., IFN-γ +874 T/A polymorphism) are a core determinant of vaccine efficacy, advocating for precision vaccinology.
6	Review	Huoming Li, Hao Li	• Detailed the distinct roles of antibody isotypes (IgM, IgD, IgG, IgA, IgE) against TB.
			• Highlighted that MTB-specific IgM in BALF was the strongest correlate of reduced bacterial burden in primates.
			• Argued that future vaccines must be designed to elicit a precisely tuned humoral immune response.
7	Correction	Yang Gong et al.	• Official corrigendum for the original research article by Gong et al. (2024).
			• Corrected errors in the labeling of figures in the supplementary materials.
			• Confirmed that the corrections do not affect any experimental data, statistical results, or the main conclusions of the study.
8	Correction	Aishwarya Shaji et al.	• Official correction for the review article by Shaji et al. (2025).
			• Restored the abstract to the authors’ originally submitted version after a production error.
			• Clarified that the correction is textual only and does not alter any data or scientific conclusions presented in the article.

### 2.1 Innovative vaccine delivery systems

Gong et al. engineered Tri-GalNAc–modified PLGA-PEG nanoparticles co-encapsulating a mycobacterial fusion protein and the STING agonist SR717 (TP/GPS). *In vivo*, Tri-GalNAc ligands enhanced dendritic cell (DC) uptake by 26% and sustained lymph node retention for over 120 hours. TP/GPS stimulated IL-12p70 secretion exceeding lipopolysaccharide levels while minimizing IL-10, inducing durable Th1/Th17 responses and tissue-resident memory T cells. In a *Mycobacterium tuberculosis* (MTB) H37Ra challenge model, TP/GPS reduced pulmonary bacterial burden comparably to BCG, validating synergistic DC targeting and innate immune activation. The accompanying correction clarified supplementary figure annotations without altering these core conclusions.

### 2.2 Diagnostic biomarkers for infection and treatment response

Ou et al. screened 24 recombinant antigens, identifying the E-M fusion protein as an optimal skin test reagent. In MTB-sensitized guinea pigs, E-M induced delayed-type hypersensitivity peaking at 24 hours (18.3 ± 1.8 mm induration) with no cross-reactivity to BCG, outperforming commercial equivalents. Preclinical toxicity studies confirmed its safety across hematological, immunological, and histopathological parameters, positioning E-M as a specific tool for LTBI detection in vaccinated populations. Conversely, Liu et al. leveraged transcriptomics to uncover three copper metabolism–related genes (ASPHD2, GK, GCH1) as diagnostic classifiers for pulmonary TB, achieving AUCs >0.92 in training and validation cohorts. These genes tracked treatment response: GCH1 and GK expression declined significantly after one month of therapy, implicating cuproptosis in TB pathogenesis and offering minimally invasive biomarkers.

### 2.3 Host-directed immunotherapy and precision medicine

Shaji et al. reviewed clinical-stage vaccine candidates (M72/AS01E, MTBVAC, H4:IC31) and highlighted that host genetic polymorphisms—such as IFN-γ +874 T/A SNPs and TOLLIP/TLR2 variants—dictate both vaccine efficacy and reactogenicity. This genetic stratification framework was mirrored by Liu et al., who found that GK expression correlated positively with neutrophils and M0 macrophages but inversely with activated CD4+ and CD8+ T cells, while ASPHD2 tracked plasma cell infiltration. The Review by Li and Li further dissected how antibody isotypes differentially control TB and implications for the vaccine development: IgM pentamers predicted bacterial clearance post-BCG vaccination, IgG1/IgG3 mediated complement fixation, IgA provided mucosal protection, and what the important scientific questions need to be answered. IgG4 responses could distinguish active from latent TB, underscoring the untapped diagnostic potential of humoral immunity.

### 2.4 TB in immunocompromised populations

Mortezazadeh et al. conducted a case-control study of 392 BCG-vaccinated individuals, finding no difference in LTBI prevalence between cancer patients and controls (27.1% vs. 20.7%). However, over four years of follow-up, cancer patients with LTBI faced a threefold higher mortality risk, and each 1-mm increase in tuberculin skin test induration was associated with a 6% increase in death hazard. This demonstrates that while malignancy does not increase LTBI acquisition, underlying immunosuppression significantly worsens clinical prognosis.

### 2.5 Scientific rigor and transparency

Two Corrections addressed figure annotation errors (Gong et al.) and an abstract text misplacement (Shaji et al.), ensuring reproducibility without compromising the robustness of the underlying science.

## 3 Discussion

The collective findings presented in this Research Topic create a powerful, synergistic narrative. They move the field forward not in isolated increments, but through a convergent evolution of ideas. For instance, the nanoparticle-based vaccine strategy by Gong et al. provides a technological answer to the immunological need for robust T-cell memory, a challenge highlighted in the vaccine pipeline review by Shaji et al. Similarly, the quest for precision permeates every layer: just as Ou et al. seek a more specific diagnostic antigen to replace crude tuberculin, Liu et al. leverage high-throughput transcriptomic data to define a precise molecular signature for TB, both efforts aiming to minimize misclassification.

Furthermore, these studies collectively argue for a holistic view of the immune response. The success of a vaccine like M72/AS01E (6, 7), noted by Shaji et al., and the engineered response by Gong et al., primarily measures T-cell-based correlates of protection. However, the review by Li and Li compellingly argues that this is an incomplete picture. The next frontier may lie in designing vaccines that not only elicit potent T-cell immunity but also guide the development of a protective antibody profile (8–13), perhaps using the very targeting technologies showcased in this topic. This integrated approach—orchestrating both arms of the adaptive immune system—is a cornerstone of modern vaccinology, as learned from COVID-19.

The clinical and diagnostic studies add another critical dimension: patient stratification. The work of Mortezazadeh et al. demonstrates that not all LTBI is equal; in an immunocompromised host, it becomes a major risk factor for mortality (14). This finding dovetails with the genetic insights from Shaji et al., suggesting that future TB control cannot be one-size-fits-all (15–17). The combination of genetic profiling, immune status monitoring (as hinted by the dynamic expression of cuproptosis genes from Liu et al.), and risk stratification (as in Mortezazadeh et al.) charts a path toward truly personalized management of TB infection and disease.

## 4 Conclusion

The collective works in this Research Topic vividly illustrate a field in rapid and sophisticated evolution. From the nano-engineering of targeted immune stimulants and the computational discovery of diagnostic biomarkers, to the refined understanding of immune correlates and the translation of epidemiological data into clinical practice, this volume showcases a multi-faceted attack on TB. The integration of key quantitative findings—from vaccine efficacy rates and diagnostic AUC values to relative risk metrics—provides a solid evidence base for future research. These advances, particularly the lessons in leveraging innate immunity and understanding host-specific responses, resonate deeply with the broader vaccinology landscape, including the fight against COVID-19. As we move forward, the continued synergy between disruptive technology, basic immunology, and precision medicine will be paramount in achieving the ultimate goal: ending the TB epidemic.
